# Study protocol for the implementation and evaluation of the Self-harm Assessment and Management for General Hospitals programme in Ireland (SAMAGH)

**DOI:** 10.1186/s12913-020-05254-x

**Published:** 2020-06-22

**Authors:** Ella Arensman, M. Isabela Troya, Sarah Nicholson, Anvar Sadath, Grace Cully, Ana Paula Ramos Costa, Ruth Benson, Paul Corcoran, Eve Griffin, Eileen Williamson, Joe Eustace, Frances Shiely, John Browne, Jan Rigby, Anne Jeffers, Eugene Cassidy

**Affiliations:** 1 School of Public Health, College of Medicine and Health, University College Cork, Western Gateway Building, Cork, Ireland; 2 National Suicide Research Foundation, University College Cork, 4.28 Western Gateway Building, Cork, Ireland; 3 Australian Institute for Suicide Research and Prevention, School of Applied Psychology, Griffith University, Brisbane, Queensland Australia; 4 Health Research Board Clinical Research Facility-Cork, University College Cork, Cork, Ireland; 5 Centre for Health Geoinformatics & Department of Geography, Maynooth University, Maynooth, Ireland; 6 National Clinical Programme for the Assessment and Management of Patients presenting to the Emergency Department following Self-Harm, Office of the National Clinical Advisor and Group Lead, Dr. Steeven’s Hospital, Dublin, Ireland; 7 Department of Psychiatry and Neurobehavioral Science, University College Cork, Acute Mental Health Unit, Cork University Hospital, Wilton, Ireland

**Keywords:** Self-harm, Suicide, Healthcare services, Suicidal intent, Process evaluation, Outcome evaluation

## Abstract

**Background:**

Previous self-harm is one of the strongest predictors of future self-harm and suicide. Increased risk of repeated self-harm and suicide exists amongst patients presenting to hospital with high-risk self-harm and major self-harm repeaters. However, so far evidence-based training in the management of self-harm for mental health professionals is limited. Within this context, we aim to develop, implement and evaluate a training programme, **SAMAGH, S**elf-harm **A**ssessment and **M**anagement Programme for **G**eneral **H**ospitals in Ireland. SAMAGH aims to (a) reduce hospital-based self-harm repetition rates and (b) increase rates of mental health assessments being conducted with self-harm patients. We also aim to evaluate the training on self-harm knowledge, attitudes, and skills related outcomes of healthcare professionals involved in the training.

**Methods/design:**

The study will be conducted in three phases. First, the SAMAGH Training Programme has been developed, which comprises two parts: 1) E-learning Programme and 2) Simulation Training. Second, SAMAGH will be delivered to healthcare professionals from general hospitals in Ireland. Third, an outcome and process evaluation will be conducted using a pre-post design. The outcome evaluation will be conducted using aggregated data from the National Self-Harm Registry Ireland (NSHRI) on self-harm repetition rates from all 27 public hospitals in Ireland. Aggregated data based on the 3-year average (2016, 2017, 2018) self-harm repetition rates prior to the implementation of the SAMAGH will be used as baseline data, and NSHRI data from 6 and 12 months after the implementation of SAMAGH will be used as follow-up. For the process evaluation, questionnaires and focus groups will be administered and conducted with healthcare professionals who completed the training.

**Discussion:**

This study will contribute to the evidence base regarding the effectiveness of an evidence informed training programme that aims to reduce repeated hospital self-harm presentations and to improve compliance with self-harm assessment and management. This study is also expected to contribute to self-harm and suicide training with the possibility of being translated to other settings. Its feasibility will be evaluated through a process evaluation.

## Background

Self-harm and suicide are major public health concerns. Worldwide over 800,000 people die due to suicide every year and is the second leading cause of death among young people [1]. For each suicide, there are more than 20 suicide attempts [1]. In 2018, the National Self-Harm Registry of Ireland (NSHRI) recorded 12,588 hospital presentations due to self-harm nationally, involving 9785 individuals [2]. Compared to 2017, self-harm hospital presentations in Ireland have increased by 7%. Furthermore, over 22% of patients repeated self-harm. Similar increasing trends have followed worldwide [3], although certain decreasing rates have been reported [4]. Furthermore, there was a significant increase in certain highly lethal self-harm methods used, such as attempted hanging, which increased by 24% from 2017 [2]. Self-harm is one of the strongest risk factors for both non-fatal self-harm repetition and suicide, with evidence indicating certain groups being more prone to repeat self-harm and/or die by suicide (e.g. men, young people, older age groups, people engaging in more lethal self-harm methods and those with a history of 4 or more previous presentations) [5, 6].

Although research indicates that most self-harm episodes occur within the community, a significant amount of presentations will occur in hospital settings, creating the opportunity for intervention when assessing and managing self-harm patients [6–8]. Evidence regarding hospital-based interventions to reduce self-harm show promising findings [7, 9, 10]. However, despite evidence indicating improved patient outcomes following assessment of self-harm patients [10, 11], many of these patients still leave hospital without an assessment [2, 12]. Moreover, the majority of hospital-based interventions for self-harm have been conducted with all self-harm patients rather than targeted subgroups [7, 9, 10]. There is increased risk of suicide among those engaging in highly lethal self-harm methods [13] and those with repeated previous self-harm attempts [14]. Previous research into these two subgroups has identified particular and distinct phenotypes for each, requiring specific interventions [15, 16]. Major self-harm repeaters have psychiatric history, including personality and mood disorder diagnoses, as well as a history of physical, emotional or sexual abuse, and method escalation. While high-risk self-harm patients have increased psychiatric and physical comorbidities, substance abuse, and are more often male. The available self-harm intervention programmes refer to the overall self-harm population, and therefore, they may not adequately address the needs of these specific subgroups. Moreover, healthcare professionals face multiple challenges in managing self-harm patients in general and high-risk subgroups in particular [17].

To date there are no evidence-based interventions for supporting these subgroups of patients. It is within this context that the SAMAGH training programme has been developed.

### Aim

In this study, we aim to implement and evaluate an evidence informed training programme, **SAMAGH, S**elf-harm **A**ssessment and **Ma**nagement Programme for **G**eneral **H**ospitals in Ireland. SAMAGH aims to (a) reduce hospital-based self-harm repetition rates overall and amongst the two subgroups and (b) increase rates of mental health assessments being conducted with self-harm patients. This study will address a number of specific objectives:

1. Assess the impact of the SAMAGH training for health professionals in hospital settings on overall repeated self-harm rates as well as in the two subgroups of high-risk patients.
2. Determine whether the SAMAGH training results in increased rates of mental health assessments being conducted with self-harm patients.
3. Conduct a process evaluation to understand the feasibility of implementing the SAMAGH training and its effect on health professionals’ knowledge, attitudes and skills on assessment and management of self-harm patients.

## Methods/ design

**Fig. 1:**
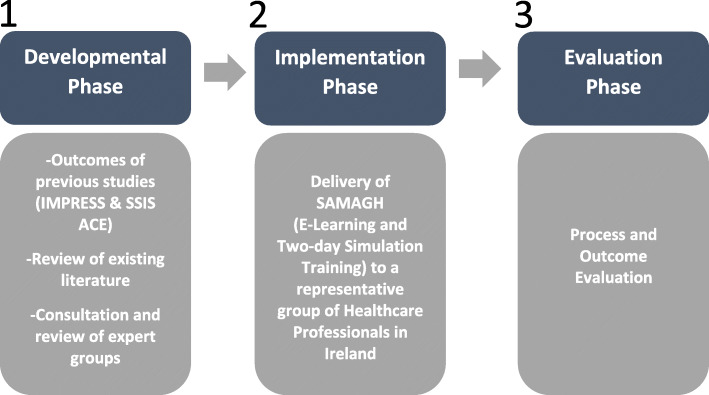
Description of the three-phase SAMAGH study

SAMAGH is a three-phase study as reflected in Fig. 1. The three methods of the phases of development, implementation, and evaluation are described in detail below.

### Phase 1: development of the SAMAGH training Programme

SAMAGH was developed based on information obtained from two previous studies on specific predictive factors for repeated self-harm and suicide among high-risk groups: SSIS-ACE (Psychological, psychiatric and work related risk factors associated with suicide in Ireland: a case control study) and IMPRESS (Improving Prediction and Risk Assessment of Self-harm and Suicide: an in-depth interview study) [18]. Extensive literature reviews, consultation with different expert groups including relevant groups of clinicians (psychiatrists, psychologists, and nurses), academics, and reviews of existing manuals for self-harm and suicide informed the development of SAMAGH.

Simulation training is a core component of SAMAGH. Simulation-based learning is a learning method that can develop health professionals’ knowledge, skills, and attitudes, whilst protecting patients from unnecessary risks [19]. Simulation training has been increasingly used in medical training, but less frequently in mental health training. It is the educational practice of recreating patient scenarios in safe environments using trained actors and technology, followed by debriefing to reinforce learning [20]. Through the use of actors, high-fidelity patient scenarios can be enacted and health professionals have the opportunity to practice and enhance the development of therapeutic alliance which is essential in mental health care [20].

We conducted an extensive review of the existing evidence of simulation training on self-harm and suicide prevention. Only five peer reviewed published studies were available, addressing self-harm/suicide related knowledge and attitude related outcomes [21–23], feasibility of the training [23], teaching learning outcomes [24], and improving gatekeeper behaviour for suicide prevention [25]. The simulation training methods varied from role-play sessions with virtual patients [23], people with lived experience of mental illness [21], simulated emergency medical scenarios with high fidelity mannequins and structured reflective debriefing [22], online role-play simulations with use of emotionally responsive avatars [25], and training using theatre students as simulated standardised patients [24]. The review did not reveal any studies that evaluated a simulation training programme specific to self-harm subgroups. Hence, we developed an evidence informed simulation-based intervention for self-harm assessment and management.

**Fig. 2:**
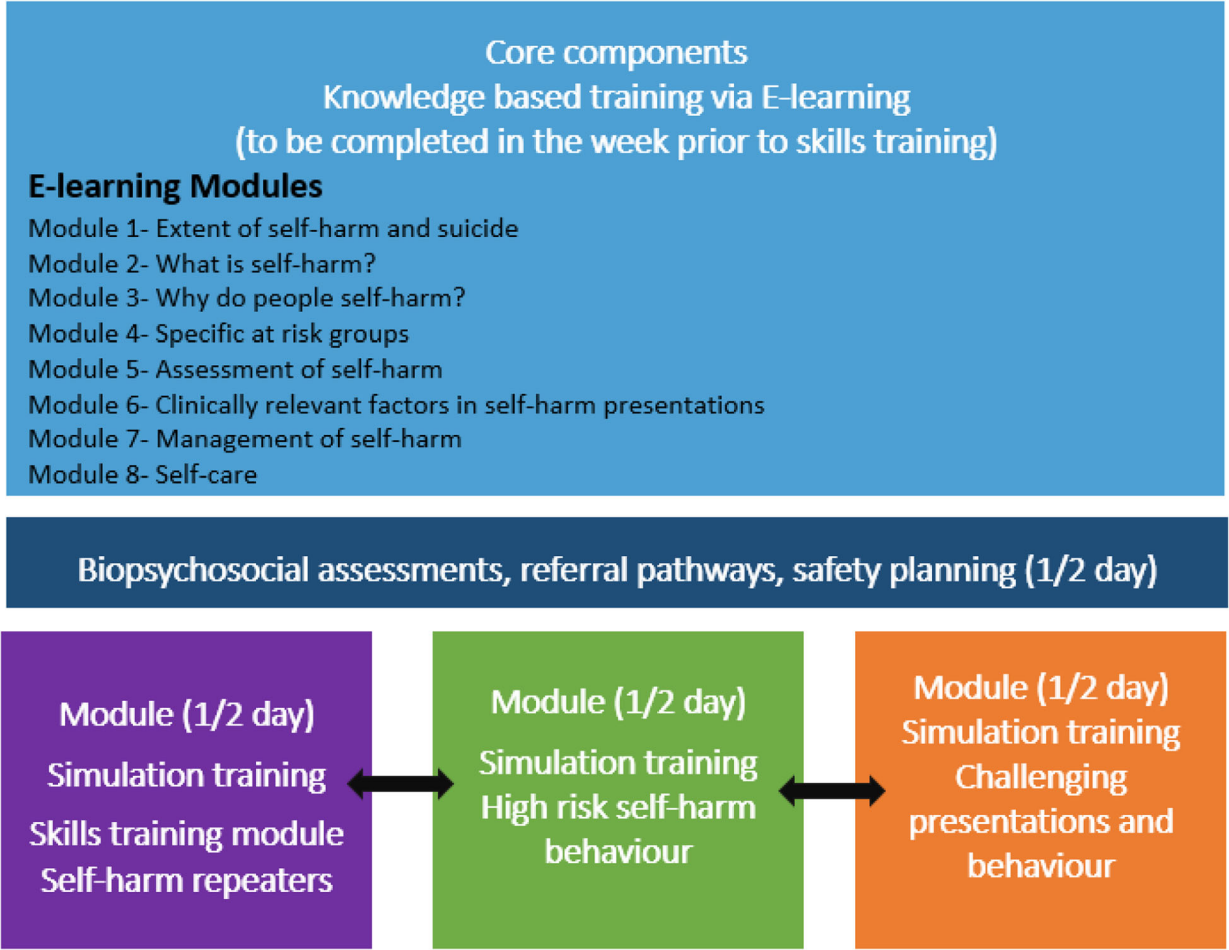
Description of SAMAGH

SAMAGH consists of two training sections. First, an E-Learning programme, where across eight comprehensive modules, health professionals will extend their knowledge of the nature and management of self-harm and suicide, with a focus on the two previously mentioned high-risk groups. The second part of the programme comprises a two-day face-to-face interactive training course, including simulation training. Figure 2 provides a description of the components of the SAMAGH training.

The subgroups of self-harm patients are defined as follows:

- Major repeater self-harm patients with a history of four or more previous self-harm presentations to hospital prior to their index self-harm presentation.
- High-risk self-harm: (a) highly lethal self-harm presentations, and/or (b) presentations with high levels of suicidal intent. The same criteria used by Arensman et al [18, 26] will be used to define high-risk self-harm, which is a combination of a highly lethal methods, medical severity and clinical impression of high suicide intent (See Additional File 1).

### Phase 2: implementation of the SAMAGH training Programme

An invitation to participate in SAMAGH will be sent to a representative group of 40 to 50 health professionals (Clinical Nurse Specialists, Non-Consultant Hospital Doctors, Psychiatry Registrars, Clinical Psychologists, Psychiatrists, among others) working across the 27 public hospitals in Ireland. Given that the SAMAGH Training Programme does not have a special focus on children, children’s hospitals will not be included in the recruitment of health professionals. Each group of health professionals will have representatives from a range of different hospitals across Ireland. Attending the SAMAGH training is not compulsory for health staff. SAMAGH will be delivered to groups of 8–10 participants, and there will be approximately five groups in total. It is expected that health professionals who complete the training will incorporate the learned skills and knowledge in their daily practice with self-harm patients.

#### E-learning Programme

One week prior to the face-to-face simulation training, participants will receive the details for the E-learning Programme via e-mail. The E-learning Programme is comprised of eight modules and will take approximately 2 to 3 h to complete. The E-learning Programme includes multiple-choice questions at the end of each module, and participants must obtain a 70% score overall to successfully complete the programme. Participants must have successfully completed the E-learning Programme prior to attending the Simulation Training as key concepts will have been introduced and reinforced to participants.

#### Simulation training

The simulation training will be held at the ASSERT centre in University College Cork. During the first few hours, participants will receive a masterclass lecture to re-inforce the knowledge from the E-learning Programme, with a specific focus on conducting a biopsychosocial assessment. A total of three patient scenarios will be given to participants for them to role play during the two-day training. The three patient scenarios include scenarios representing high-risk self-harm patients, major self-harm repeaters, and challenging behaviour patients. Participants will learn how to best assess and manage these self-harm patients through simulation training. The three patient scenarios will be played by actors who have had previous experience in medical simulation training and who received in-house inductions on the different patient roles to be played.

Facilities from the ASSERT centre include two rooms which simulate a hospital Emergency Department setting and have two-way mirrors (see Additional File 2). Participants will be divided into groups of two, in order to optimise learning in smaller groups. All participants will be individually involved in the simulation training, playing out the three patient scenarios each. The remaining participants will observe and learn from their colleagues who are completing the simulation training, and will be accompanied by facilitators/reviewers, who will grade participant’s simulation training. At the end of each simulation training, the facilitators/reviewers will provide feedback to the participant and group, making it a group learning exercise. Facilitators for the simulation training include self-harm and suicide prevention experts, including consultant psychiatrists, academic professors and the national clinical lead and head of nursing for the national clinical programme of self-harm assessment and management of patients presenting to emergency departments.

### Phase 3: evaluation of the SAMAGH training Programme

**Fig. 3:**
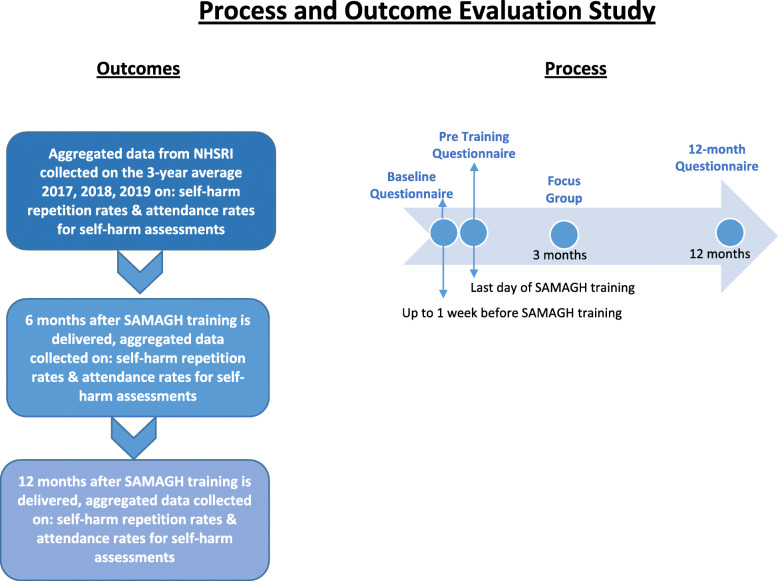
Description of the Process and Outcome Evaluation Study

SAMAGH will be evaluated through a process and outcome evaluation study as described in Fig. 3. Further description of the study design is presented in the next section.

#### Process evaluation

Health professionals who completed the training will take part in questionnaires and focus groups. The United Kingdom’s Medical Research Council (MRC) guidelines for evaluating complex interventions will be followed for the process evaluation [27]. Levels of adherence to the SAMAGH training programme will be assessed via the process evaluation.

All health professionals taking part in SAMAGH will be invited to take part in all three questionnaires (See Additional File 3). The pre-training questionnaire will measure health professionals’ current knowledge, attitudes and confidence relating to self-harm assessment and management, prior to attending the SAMAGH training. The baseline questionnaire to be conducted at the end of Day 2 of the SAMAGH simulation training, will measure: a) the acceptability of SAMAGH as an intervention to reduce hospital self-harm repetition rates and increase rates of mental health assessments being conducted with self-harm patients, b) gained knowledge on self-harm assessment and management. An online follow-up questionnaire will be conducted 12 months after receiving the training where the level of adherence to SAMAGH will be measured, as well as knowledge, attitudes and confidence on self-harm assessment and management. All questionnaires will be self-report, anonymous, and include a mix of true or false, open ended, multiple choice and Likert questions.

The sample of the focus groups will consist of 15 to 20 randomly selected health professionals attending the SAMAGH training programme. Randomisation of participants for the focus group will be done using a computer-randomised algorithm. Two focus groups will be conducted in total, approximately 3 months after the SAMAGH training, with 8–10 participants in each focus group. A topic guide will be used for the focus groups in order to obtain health professionals’ experiences supporting patients presenting with self-harm behaviour and existing challenges faced when offering support. Specific questions around observation of any changes for recurrence rates, and usefulness of the SAMAGH training programme will also be made. The focus groups will be conducted by specially trained researchers and will last approximately 2 h. Focus groups will be audio-recorded (if all participants agree to be recorded) and consent will be obtained for this data collection procedure. All participants will provide written informed consent prior taking part in the study.

#### Outcome evaluation

An outcome evaluation study will be conducted using a pre-post design. NSHRI aggregated data at a national level across the 27 hospitals in Ireland will be used, based on the 3 year average before the implementation of the SAMAGH training programme and 6 and 12 months after implementation of the programme. Outcomes to be included are: (a) hospital self-harm repetition rates, and (b) assessment rates following the hospital self-harm presentation. Aggregated data from the NSHRI allows for the analysis of hospital self-harm repetition rates of subgroups of self-harm patients, including patients with a history of 4 or more previous hospital self-harm episodes and those presenting to hospital following high-risk self-harm.

#### Statistical analysis

IBM SPSS 26 data software manager will be used to perform quantitative data analysis. The analysis for the pre-post evaluation study will focus on two outcome variables: (a) changes in hospital self-harm repetition rates, overall and in the two subgroup of high-risk patients, and (b) changes in rates of mental health assessments being conducted with self-harm patients. To analyse changes in the overall hospital self-harm repetition rates, Poisson regression will be used to compare the mean proportions of self-harm repetition rates at baseline, 6 and 12 months follow-up, using a moving window. This analysis will also address differences between the subgroups of major self-harm repeaters and high-risk patients. Hospital self-harm assessment attendance rates at baseline, 6 and 12 months follow-up will also be analysed using Poisson regression. Adjustment of relevant variables, such as services availability, type of hospital, staff trained, etc., will be made during the analysis.

Thematic analysis will be used in order to analyse qualitative data from the focus groups [28]. Data analysis software manager QSR NVivo 12 will be used to assist in the analysis of the dataset, which consists on transcripts from the focus groups.

#### Sample size and power calculation

Using 2017 as an example, there were 11,600 hospital-based self-harm presentations, involving 9103 individuals, across the 27 hospitals in Ireland [2]. The proportion of acts accounted for by repetition in 2017 was 21.5%. Of the 9103 self-harm patients treated in 2017, 1322 (14.5%) made at least one repeat presentation to hospital during the calendar year [2]. It is expected that similar numbers will arise from the 3-year average rates from 2016, 2017, and 2018. In order to achieve the anticipated reduction from 12 to 8% reduced self-harm repetition rate, which will be detected by achieving 90% power at 0.05 significance level, it is expected that enough cases will be identified throughout the 27 hospitals collected at baseline and at 6 and 12 months follow-up, following patterns of data from 2017.

### Data management and access

The protocol complies with the Irish Data Protection Act of 1988 and the Irish Data Protection Amendment Act of 2003 and General Data Protection Regulation (2018). Only anonymised data will be released in aggregate form in academic journal articles, conference papers, and reports.

The Pre Training questionnaire and the 12-month online questionnaire will be conducted using the E-learning Website and Survey Monkey, which can be completed without providing any contact or personal information.

Existing protocols will be followed to ensure maintaining patient confidentiality. No details that would allow identification of participants will remain anywhere but in the consent forms, which will be securely stored.

The hard data from focus groups will be assigned ID numbers and the code key of participants’ names will be kept in a separate locked cabinet. Electronic data will be password-protected and stored on an encrypted computer within offices to which only NSRF staff have swipe access. Participants will be permitted access to their personal data at any time and will be entitled to withdraw their data from the study up to the point of publication. Manual data and electronic data will be stored securely in locked cabinets and encrypted computers within the NSRF offices for 10 years after collection.

### Precautions

The researchers involved are experienced in the area of mental health research and have received specialised in-house training in relation to responding to distress and risk in the context of face-to-face contact. The researchers will receive weekly supervision from the Principal Investigator (PI), Professor Arensman.

Participants will be fully informed of the aims and procedure of the research and researchers will be mindful of potential risk. Specific arrangement for participants will be made if required. Participants can consult with the PI, Professor Ella Arensman. Interviewer safety will be taken into account at all times. Debriefing sessions will be held with the PI (a trained psychotherapist, Professor Ella Arensman) and the involved researchers on a weekly basis or more regularly if required.

## Discussion

Interventions aimed at reducing hospital-based self-harm presentations and hospital support received are crucial for self-harm patients and society. Identifying and managing high-risk hospital self-harm presentations is key for prevention of repeated self-harm and suicide [1]. Repeated self-harm presentations remain an ongoing challenge in hospital settings, both in Ireland and globally [1–5]. A numerable amount of such hospitalisations could be prevented by improving care and support given to self-harm patients presenting to hospital. To our knowledge, this is the first study to address improving care and support given to the clinical subgroups of at risk self-harm patients: major self-harm repeaters and high-risk self-harm (HSRH) patients, as well as evaluate the training programme through a process and outcome evaluation. In addition to a direct contribution to health care at a national level, it is expected that this study will contribute to the development of suicide prevention and intervention programmes. This study is also expected to contribute to self-harm and suicide training capacity building activities, with the possibility of being translated to other settings.

## Supplementary information

**Additional file 1.** Identification of highly lethal self-harm. Table summarising identification of highly lethal self-harm.

**Additional file 2.** Facilities of the ASSERT centre. Photographs of simulation labs used in the training at the ASSERT centre.

**Additional file 3.** Baseline Questionnaire SAMAGH. Baseline Questionnaire used as part of process evaluation.

## Data Availability

Not applicable.

## Abbreviations

HRB: Health Research Board
HSRH: High-risk self-harm
IMPRESS: Improving Prediction and Risk Assessment of Self-harm and Suicide
MRC: Medical Research Council
NSHRI: National Self-Harm Registry of Ireland
NSRF: National Suicide Research Foundation
SAMAGH: Self-harm Assessment and Management programme for General Hospitals
SSIS-ACE: Psychological, psychiatric and work related risk factors associated with suicide in Ireland
UCC: University College Cork
