# Supplementary material for: Study protocol for the implementation and evaluation of the Self-harm Assessment and Management for General Hospitals programme in Ireland (SAMAGH)
Source: BMC Health Serv Res. 2020 Jun 22;20:590. doi: 10.1186/s12913-020-05254-x (PMC7322837; doi:10.1186/s12913-020-05254-x)
Supplement: Supplementary file 1 — Additional file 1. Identification of highly lethal self-harm. Table summarising identification of highly lethal self-harm. [file 12913_2020_5254_MOESM1_ESM.docx]

# Supplementary Material

## Additional File 1. Identification of highly lethal self-harm

File name: Appendix 1 Identification of highly lethal self-harm

Title of data: Appendix 1 Identification of highly lethal self-harm

Description of data: Table summarising identification of highly lethal self-harm

| Method | Considered highly lethal |
| --- | --- |
| *Attempted hanging*  *Ligature use*  *Self-strangulation* | *Unconscious after attempting to hang or use a ligature, or not unconscious but:*   1. *Witnessed in suspension or using a ligature and physical evidence of asphyxiation; or* 2. *Physical evidence of suspension or using a ligature* |
| *Self-asphyxiation*  *Suffocation* | *Witnessed self-asphyxiation, or any other physical evidence of self-asphyxiation* |
| *Cutting*  *Stabbing*  *Wound aggravation*  *Or insertion* | *Sustained a puncture wound penetrating body cavity or major organ, or lacerations that damaged or severed tendons, arteries or large veins, or came very close to doing so. Treatment in specialised unit i.e. intensive care.*  *Surgery under general anaesthesia* |
| *Ingesting, inhaling, injecting*   1. *Level of consciousness* 2. *Biochemical abnormalities* | 1. *Objective evidence of altered level of consciousness, or unconscious at presentation or prior to medical facility* 2. *Admission to intensive care unit, hyperbaric unit.* 3. *Extensive medical treatment including antidotes for drug overdoses, telemetry, or repeated tests or investigations* |
| *Jumping* | *Witnessed jumping or any physical evidence of having jumped from a considerable height, likely to have led to serious injury* |
| *Other* | *Determined on a case by case basis* |
