# Supplementary material for: Study protocol for the implementation and evaluation of the Self-harm Assessment and Management for General Hospitals programme in Ireland (SAMAGH)
Source: BMC Health Serv Res. 2020 Jun 22;20:590. doi: 10.1186/s12913-020-05254-x (PMC7322837; doi:10.1186/s12913-020-05254-x)

## Additional File 2. Facilities of the ASSERT centre

File name: Additional File 2 Facilities of the ASSERT centre

Title of data: Additional File 2 Facilities of the ASSERT centre

Description of data: Photographs of simulation labs used in the training at the ASSERT centre


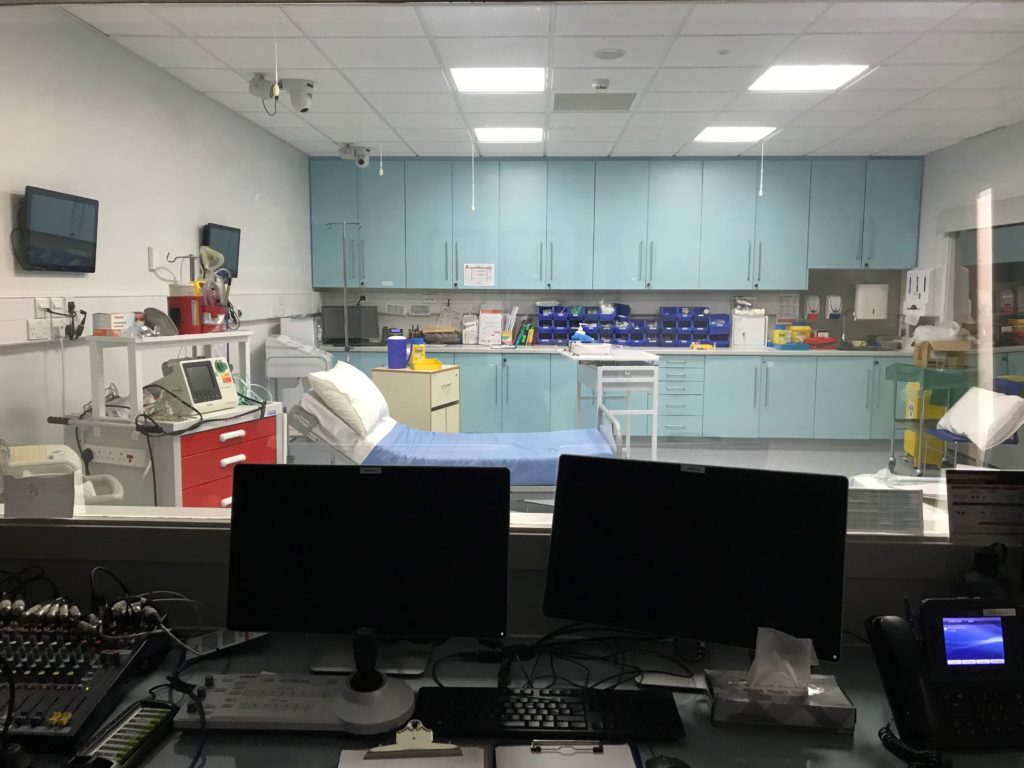


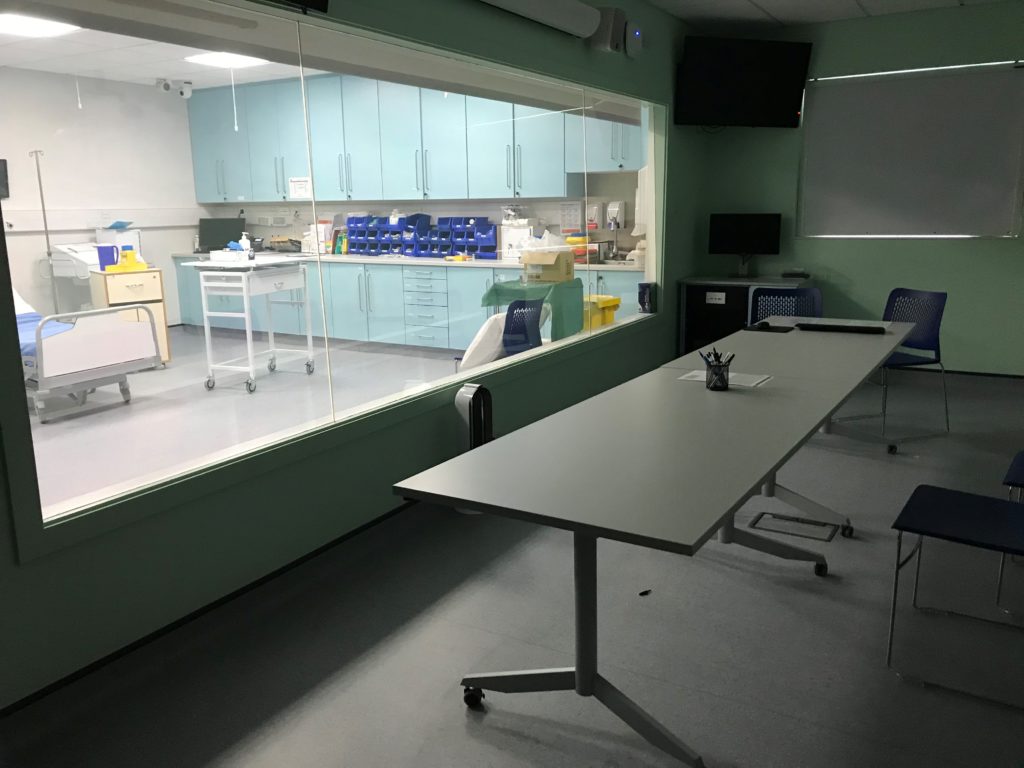

Supplement: Supplementary file 2 — Additional file 2. Facilities of the ASSERT centre. Photographs of simulation labs used in the training at the ASSERT centre. [file 12913_2020_5254_MOESM2_ESM.docx]
