# Supplementary material for: Study protocol for the implementation and evaluation of the Self-harm Assessment and Management for General Hospitals programme in Ireland (SAMAGH)
Source: BMC Health Serv Res. 2020 Jun 22;20:590. doi: 10.1186/s12913-020-05254-x (PMC7322837; doi:10.1186/s12913-020-05254-x)
Supplement: Supplementary file 3 — Additional file 3. Baseline Questionnaire SAMAGH. Baseline Questionnaire used as part of process evaluation. [file 12913_2020_5254_MOESM3_ESM.docx]

## Additional File 3. Baseline Questionnaire SAMAGH

File name: Additional File 3 Baseline Questionnaire SAMAGH

Title of data: Additional File 3 Baseline Questionnaire SAMAGH

Description of data: Baseline Questionnaire used as part of process evaluation

**Self-Harm Assessment and Management Survey: Baseline Questionnaire**

This survey is part of an evaluation study being undertaken by University College Cork (UCC), and the National Suicide Research Foundation, supported by the Health Research Board. Research staff from this project will be collecting the survey results. You are invited to take part in this survey because we want to understand your experience and views after receiving the **S**elf-Harm **A**ssessment and **Ma**nagement for **G**eneral **H**ospitals Training Programme (**SAMAGH**). Participation in this survey is voluntary and anonymous. If you choose to take part, you may change your mind and stop the survey at any time. This survey should take approximately 10 minutes to complete. Information from this survey will help us understand your views on the recently received SAMAGH training programme. **Thank you for your participation!**

1. Date (mm/dd/yy )____________________
2. I am a: Clinical Nurse Specialist Clinical Psychologist Psychiatrist
   Non Consultant Hospital Doctor Psychiatry Registrar Other ______________
3. Identify the sections you found most beneficial after receiving this week’s training.

| **Sections** | **Not beneficial** | **Hardly beneficial** | **Indifferent** | **Somewhat beneficial** | **Extremely beneficial** |
| --- | --- | --- | --- | --- | --- |
| Online Module 1: The extent of self-harm and suicide |  |  |  |  |  |
| Online Module 2: Evidence informed assessment |  |  |  |  |  |
| Online Module 3: Dealing with self-harm patients |  |  |  |  |  |
| Online Module 4: Subgroups of people who self-harm |  |  |  |  |  |
| Online Module 5: Self-care |  |  |  |  |  |
| Simulation training major repeaters |  |  |  |  |  |
| Simulation training high-risk self-harm patients |  |  |  |  |  |

**For the questions below, check the box that best fits your opinion:**

1. Overall, I feel confident about providing assessment and management to self-harm patients after receiving today’s training.

Strongly Disagree Disagree Agree Strongly Agree

1. Overall, I feel I will be able to deliver to patients the learned assessment and management programme.

Strongly Disagree Disagree Agree Strongly Agree

1. Overall, I feel there are the available resources and infrastructure to deliver SAMAGH.

Strongly Disagree Disagree Agree Strongly Agree

7. Overall, I think SAMAGH will have:

positive effect no effect negative effect **on all self-harm patients**

positive effect no effect negative effect **on patients with repeat self-harm**

positive effect no effect negative effect **on my ability to offer patient
 support**

8. Do you plan to use the self-harm assessment and management training programme in your patients following the training?

Yes No Undecided

9. Do you foresee any obstacles for the implementation of SAMAGH?

Yes No

If you answered “yes”, please describe:

10. If you do not plan using SAMAGH to assess and manage self-harm patients, why not?

Not enough time Does not seem appropriate

Lack of institutional support The content is too difficult to deliver

Unrealistic with patients Do not think it will make a difference

Other – *Please specify*

***THANK YOU FOR TAKING PART IN THIS SURVEY***
